# Cook County Medical Society

**Published:** 1852-06

**Authors:** 


*Cook Co. Med. Society.*

May 4th. The Society met, the President, Dr. McArthur, in the chair. An Essay was read by Dr. J. W. Freer, on "the Nature and Pathology of Rheumatism," from which we make the following extracts :—

"The fever has been considered as a natural element of the disease and not adventitious, as I shall maintain is the case, and for the following reasons:

"The first argument which may be offered in support of this proposition is, that the disease in the acute form is almost uniformly ushered in by the local symptoms, and the fever which follows seems to be the consequence of the local irritation rather than an original constitutional affection; furthermore, this fever subsides long before the local manifestations have ceased to operate, in fact the local symptoms are frequently or generally aggravated after the cessation of the fever.

"I do not wish to be understood as upholding the theory that this disease is entirely local in its nature, but that there is a common pathology for each variety of this affection, and that febrile excitement is not a necessary constituent but a complication, while the condition of the blood, viz., an excess of the fibrinous element, which always maintains in this variety of fever, is not to be reckoned among the morbid evidences and pathological conditions of rheumatism.

"In regard to the idea of a rheumatic diathesis, or any other diathesis existing in an individual who is in the enjoyment of perfect health, all the functions performed normally, it seems to me to be untenable, and can subserve no good purpose, in fact can answer no end but that of bolstering up theories.

"Should the kidneys or other organs of depuration, in case of a suppression of the cutaneous exhalation, be insufficient for the performance of the extra labor imposed upon them, we must then have an accumulation of morbid products in the vascular system, by which the wheels of life must be more or less clogged. The nicely adjusted balance between the functions of assimilation and re-production on one hand, destruction and depuration on the other, is destroyed.

"The system is now in a condition to take on diseased action, and the force which determines the kind must, in the present state of our knowledge, remain undetermined.

"The joints, which are the regions usually selected, are so constituted, anatomically, that they are least capable of self-protection against atmospherical vicissitudes. For instance, the soft tissues surrounding the joints, the white fibrous, are those possessing the lowest grade of organization and vitality, besides being scanty in bulk. So much is this the case, that the bony constituents of these regions are in a great measure subcutaneous, and the aggregate amount of blood circulating in these parts is much below that of the adjacent regions, which are principally muscular. We are all aware, from experience, how much sooner than others these parts suffer from the effects of cold which are supplied with little or no muscular tissue, viz., the fingers, back of the hands, feet, knees, &c., while the adjacent muscular regions maintain a comfortable condition of temperature.

" From these facts we may infer that there is a lack of capability in these parts to maintain a normal standard of temperature under adverse circumstances. Moreover, a reduction of the normal standard of animal heat being equal to a reduction of vitality and vital force, we may readily predict that those regions which are subject to these conditions are necessarily more obnoxious to interference and derangement from the above causes than others which are differently constituted.

"Furthermore, we may conclude from these statements, that with an individual in sound health (in fact, without a diathesis for this or any other malady,) these may be considered as among the weaker points of his physical constitution, and may determine the species of the diseased action.

" From the known phenomena attending the introduction of this disease, that is its apparently local nature, manifesting a decided preference for parts of a definite structure—the joints—it follows that the affection, in the onset, is local in its character; but this does not preclude the idea of a general pathological condition ensuing, which may be peculiar and characteristic."

In regard to the lactic acid theory, the Doctor remarks: " From all the testimony which I have been able to gather, not a single point of direct evidence appears to establish the presence of lactic acid in blood, either in health or disease, more than the theoretical and unqualified assertions of certain authors and experimenters. That it is not a normal constituent of the blood has been well proven by Leibig, although he found it as an ingredient of the juices of the flesh, either as constituent *per se* or the result of chemical decomposition in the analysis.

"The theories in regard to the uric acid pathology of rheumatism are established upon observations made in the acute variety of the disease. The fever which attends being analogous to all fevers of an acute or sthenic character, in all of which we find this same excess of uric acid in the urinary secretions. In regard to the presence of uric acid in the blood, Ganvel has proven by experiments and analysis upon the blood of rheumatic patients, that it contains no more uric acid than in health, while the contrary maintains in the gouty conditions.

"In conclusion, I would offer the query, whether there is anything like a natural limit to the duration of acute rheumatism? If not, than we are unable to decide, with any degree of accuracy, as to the real benefits derived from any of the therapeutical doctrines in vogue, and shall never be able to arrive at correct conclusions until we are able to confine the disease to a limited and comparatively brief space of time."

Dr. Herrick remarked, that while the doctor attempts to overthrow theories he proposes one of his own, he assumes a want of balance between primary and secondary assimilation. We may learn much from an analysis of the secretions and of the blood in this disease, by which we shall be enabled to determine whether acids, alkalies, or neutral salts are indicated. Two cases, now in my mind, illustrate the benefit from the use of the organic acids.

1st. That of a boy about 16—for six months had only been able to get about with difficulty. Had been under homœopathic treatment. He was directed to eat three lemons daily, in three days he came back without his cane; directed to continue, in three days more was perfectly well. 2nd. The case of a sailor, who had been unable to do anything for six months—much pain and swelling in his ancles—every indication of severe inflammatory rheumatism. He was ordered lime juice ℥js three times daily, with grs. x. bi-carb. potass. In about ten days he was discharged well.

Dr. Cheeny had usually adopted the antiphlogistic treatment, calomel, opium, antimony, bleeding, and the protection of the diseased parts. Has generally found the action of the heart increased; reduce this and the disease soon gives way.

Dr. McArthur has had some experience personally in this disease, doubts whether the idea that perspiration is suppressed at the commencement of rheumatic attacks is correct. Has noticed a change in feeling without observing any change in the condition of the skin; thinks the nice appreciation, by rheumatic patients, of atmospheric changes, important in studying the pathology of the disease. Has heard of chloroform being used, does not know with what success. Has seen benefit result from the use of the organic acids.

Dr. Johnson thought that the fact of the disease originating in the white fibrous tissues surrounding the joints—parts possessed of low vitality, and peculiarly susceptible to atmospheric changes,—seems to indicate that the first step in the morbid process is the formation, instead of urea of uric acid, which, combining with the earthy bases, forms a deposit giving rise to pain.

Dr. Bird thought the different methods of treatment might all be beneficial by accomplishing one object, namely, the prevention of excessive oxydation; opium in full doses reduces the number of respirations, and thus prevents the introduction of oxygen; the organic acids, on the other hand, neutralize it after it is introduced; the antiphlogistic treatment by depletion, antimony, &c., has the same ultimate effect as opium, viz., to lessen the amount of oxygen taken into the system. J.

